# Illness perceptions mediate the relationship between bowel symptom severity and health-related quality of life in IBS patients

**DOI:** 10.1007/s11136-015-0932-8

**Published:** 2015-02-07

**Authors:** Véronique De Gucht

**Affiliations:** Health Psychology Unit, Faculty of Social Sciences, Institute of Psychology, Leiden University, Wassenaarseweg 52, PO BOX 9555, 2300 RB Leiden, The Netherlands

**Keywords:** Irritable bowel syndrome, Illness perceptions, Disease-specific health-related quality of life, Symptom severity

## Abstract

**Purpose:**

Irritable bowel syndrome (IBS) is a functional bowel disorder with a large negative impact on HRQOL. The present study examines whether severity of bowel symptoms is directly related to HRQOL, and/or indirectly, mediated by the patients’ illness perceptions.

**Methods:**

Patients were recruited from an IBS support group (*N* = 123), and data were collected online. HRQOL was measured with the Quality of Life Measure for Persons with IBS and illness perceptions with the brief Illness Perception Questionnaire. Mediation models were tested using the bootstrapping procedure developed by Hayes.

**Results:**

Irritable bowel syndrome symptom severity is directly related to total HRQOL and its subscales; after entering the mediator variables (i.e. the patients’ illness perceptions) into the model, this direct association remained only significant for total HRQOL. The relationship between bowel symptom severity and total HRQOL was partially mediated by illness perceptions, and its relationship with each of the HRQOL subscales was fully mediated by the patients’ illness perceptions. Perceived consequences were a mediator of the relationship between bowel symptom severity, total HRQOL as well as its subscales, with the exception of Sexuality.

**Conclusions:**

Bowel symptom severity not only has a direct relationship with HRQOL, but also an indirect relationship via the patients’ cognitive and emotional representations of their illness. In order to better understand this relationship, future research should not only include illness perceptions but also assess cognitive and behavioural coping responses. Clinicians wanting to improve patients’ HRQOL should not only focus on the patients’ symptoms, but also on their illness beliefs and coping responses.

## Introduction

Irritable bowel syndrome (IBS) is a functional disorder of the bowel, meaning that it is not characterized by any structural abnormality, but rather by unexplained pain and bowel dysfunction [1]. It is diagnosed by the “Rome III” criteria [2]: abdominal discomfort or pain which is (a) relieved by defecation and is (b) associated with a change in stool frequency and/or consistency and/or (c) associated with a change in the form or appearance of stool. For a patient to meet these diagnostic criteria, the symptoms and associations must have been present at least 3 days per month for the past 3 months, with the initial onset of symptoms 3 months or more in the past.

The prevalence of IBS in the general population is high, ranging, depending upon the study, from 3 to 32 %, with most studies reporting a prevalence of between 5 and 15 % [3]. The overall prevalence found in a large survey of 40,000 subjects from different European countries was 11.5 % [4]. A population study conducted in the USA revealed an overall prevalence of 14.1 % [5]. With respect to gender differences, there is a higher ratio of women who develop IBS compared to men, ranging from 2:1 to 3:1 [6, 7]. The prevalence of IBS decreases slightly with age [3]. The socio-economic impact of IBS, based on direct medical costs, decreased work productivity and increased work absenteeism, is substantial [7, 8]. A recently published review reported that the direct medical costs associated with IBS range, depending upon the study, from 1,562$ to 7,547$ per patient per year [9].

Next to a socio-economic impact, IBS also has a considerable impact upon the patients’ personal lives and more in particular upon their health-related quality of life (HRQOL). HRQOL encompasses the appraisal of a patient regarding his or her physical or mental health. This appraisal depends upon whether patients experience limitations due to their disease and/or the extent to which their disease interferes with their life and overall functioning [10]. Despite the fact that IBS is not a life-threatening disease, the syndrome has a large negative impact on HRQOL, when compared to healthy controls [7, 8, 11–13]. Several studies have also shown that HRQOL in IBS is equal to or lower than QOL in patients suffering from other chronic diseases and conditions including inflammatory bowel diseases, asthma, migraine, gastro-esophagal reflux disease, diabetes, dialysis-dependent end-stage renal disease, breast cancer, heart disease and severe obesity [10, 14–18].

With respect to the factors influencing HRQOL in IBS, empirical studies point at severity of bowel symptoms and psychological factors as the main determinants [8, 14, 19–21]. A study by Naliboff et al. [22] showed that psychological factors had even a stronger direct effect on HRQOL than bowel symptoms. In addition, bowel symptoms were found to also exert an indirect effect on HRQOL, mediated by psychological distress. With respect to the physical and the mental components of HRQOL, there seems to be a differential pattern of association: severity of bowel symptoms was found to be a stronger predictor of physical functioning, whereas psychological factors such as anxiety and depression were found to be stronger predictors of psychological functioning measured by the SF-12 and SF-36 [23, 24]. This does not come as a surprise as the psychological functioning dimension of HRQOL scales such as the SF-12 and SF-36 include items that could also be part of anxiety and depression scales. It is therefore important to look at possible psychological sources of anxiety, depression and HRQOL in IBS patients.

From this perspective, the beliefs that patients hold about their illness and treatment are potentially important determinants. Leventhal et al. [25] developed the common sense model (CSM) classifying these beliefs or illness perceptions as follows: identity (the label given to and the symptoms associated with the illness); timeline (beliefs about duration and course of the illness); consequences (perception of the illness’s effects on the patient’s daily life and functioning); causes (the patient’s belief about the likely cause(s) of the disease) and control (the amount of control the patient feels he/she has over the illness and the extent to which he/she considers the medical treatment to help). A meta-analysis [26] demonstrated a link between illness perceptions measured according to Leventhal’s CSM and HRQOL as well as psychological well-being across a range of different illnesses. More specifically, perceptions that the illness was controllable were positively related to psychological well-being, social functioning and vitality and negatively related to psychological distress. Perceived consequences, timeline and identity on the other hand were negatively related to psychological well-being, social functioning and vitality.

Only a few studies have investigated the relationship between illness perceptions and HRQOL in IBS. In their study, Rutter and Rutter [27] found that members of an IBS patient support network that perceived a lot of consequences of IBS and attributed their symptoms to a psychological rather than a physical cause reported lower HRQOL and higher anxiety and depression scores. Perceiving little control over the illness and thinking that IBS would be difficult to cure was associated with both lower HRQOL and higher depression scores. In another study, adopting a longitudinal perspective, Rutter and Rutter [28] found that strong consequence beliefs at baseline predicted higher anxiety and depression scores at 12-month follow-up in a population of IBS patients consulting their primary care physician. A study by Riedl et al. [29] demonstrated that IBS patients with mainly somatic illness attributions reported worse physical HRQOL, whereas patients with mainly internal psychological attributions reported better physical but worse mental HRQOL.

While the aforementioned studies pointed at a relationship between illness perceptions and HRQOL in IBS, in each of these studies HRQOL was measured by means of a generic rather than a disease-specific questionnaire. Generic measures typically assess HRQOL independent of the specific characteristics of a particular disease, making them suitable for comparing HRQOL across diseases. The disadvantage of a generic measure is, however, that it is not condition-specific, and as a consequence, important aspects related to the impact of a particular disease on HRQOL may be neglected. For the purpose of the present study, a disease-specific HRQOL measure was used. This allows us to capture the patients’ subjective evaluation of HRQOL more adequately as it specifically addresses the patients’ concerns and experiences associated with the symptoms of IBS [30].

Previous studies on the relationship between illness cognitions and HRQOL have not taken the severity of IBS symptoms into account. A review article on HRQOL in IBS concluded, however, that “the severity of the bowel symptoms in IBS patients is associated with a corresponding impact on HRQOL” [14, p. 118]. It is therefore important, when studying the relationship between patients’ cognitions about their condition and HRQOL, to also include the severity of bowel symptoms as a potential determinant of HRQOL. The study by Naliboff et al. [22], pointing at the fact that bowel symptom severity has not only a direct effect on HRQOL, but also an indirect effect, mediated by psychological factors, suggests that a mediation model is an adequate way to study the association between symptom severity, cognitive factors (i.e. illness perceptions) and HRQOL in IBS patients.

For the purpose of the present study, it was hypothesized that patients’ illness perceptions mediate the impact of bowel symptom severity on HRQOL. The direction of the pathways was based upon the core principle/basic tenet of cognitive behavioural theory, namely that an event or trigger (i.e. bowel symptoms/pain) sets in motion cognitive processes (i.e. negative beliefs/illness perceptions), leading to negative emotional or behavioural consequences (i.e. poor HRQOL) [31]. This core principle has also been integrated in explanatory models for medically unexplained symptoms and syndromes [32].

### Research question

The present study examines to what extent severity of bowel symptoms is directly related to disease-specific HRQOL in IBS patients, and/or indirectly, mediated by the patients’ illness perceptions. It is also examined whether there is a differential pattern of associations between severity of bowel symptoms, illness perceptions and HRQOL depending upon the specific (physical, emotional or social) dimension of HRQOL that is looked at.

## Methods

### Study design and procedure

Data were collected with an online self-report measure. Respondents were recruited from an IBS patient support group by placing an advertisement and an invitation to participate on the Internet forum and newsletter of the support group. If patients were interested in participating in the study, they could log on to the website anonymously. On the website, a letter containing information on the study was provided, accompanied by a statement emphasizing the confidentiality of data collection. Respondents gave their informed consent by ticking a box before they got access to the study questionnaire. The study was approved by the ethical committee of Leiden University.

### Participants

One hundred and forty-four subjects filled in the questionnaire (126 women and 18 men). Subsequently, 21 subjects were excluded from the study because they did not qualify for an IBS diagnosis according to the Rome III criteria [2]. The final sample consisted of 123 subjects (108 women and 15 men). Ages ranged from 16 to 61, with a mean age of 32.43 (SD = 11.82). The female-to-male ratio in the present study (88 vs. 12 %) is not in accordance with existing epidemiological data [6, 7]. It is, however, comparable to the ratio reported by Rutter and Rutter [27], a study that also recruited respondents from an IBS network.

### Measures

A self-report questionnaire based upon the “Rome III” criteria for IBS was administered to determine whether the respondents qualified for an IBS diagnosis. The questionnaire contained 16 items, measuring different IBS symptoms such as bowel pain, stool frequency and stool consistency, based upon the Rome II Modular Questionnaire [33] and adapted to fit the newer “Rome III” criteria.

Irritable bowel syndrome symptom severity was measured with three questions from the irritable bowel severity scoring system (IBSSS) [34], measuring (a) severity of abdominal pain (0 = no pain, …, 100 = severe pain), (b) severity of abdominal distension or bloating (0 = no distension, …, 100 = severe distension) and (c) number of days patients experienced pain in the last 10 days. A total severity score was calculated by adding up the score on (a), the score on (b) and the number of days (c) multiplied by 10, leading to a possible total score of 300. Francis et al. [34] examined the reliability and validity of the IBSSS. The test–retest reliability of the IBSS was good as scores repeated within 24 h were highly reproducible. The IBSSS was also found to distinguish well between healthy controls and patients suffering from IBS, and between patients clinically diagnosed with mild, moderate and severe IBS, indicating that the criterion validity of the IBSSS was good. Finally, the responsiveness (sensitivity) to change was found to be very good; in patients who became considerably better, the severity score showed a highly significant improvement.

Illness perceptions were measured using the Dutch Language Version of the Brief Illness Perception Questionnaire (Brief IPQ-DLV) [35] which has been shown to have acceptable reliability and validity. The Brief IPQ was shown to have good test–retest reliability over a period of 3 and 6 weeks. The questionnaire also demonstrated to have good concurrent validity with similar measures and to adequately predict a number of relevant disease outcomes such as return to work and HRQOL across different patient groups [36, 37]. The (single-item) dimensions consequences, timeline, identity, personal control, treatment control and coherence, and the (two-item) dimension emotional representation were administered. One open-ended question measures the perceived cause of the illness. Respondents had to note down what they considered to be the most important cause of their IBS. Subsequently, these answers were recoded into a dichotomous variable where 1 = a psychological cause (e.g. stress or life events) and 2 = a somatic cause (e.g. heredity or immune deficiencies). For the subscales consequences, timeline, identity and emotional representation, higher scores represent more negative illness perceptions (e.g. more consequences or a longer timeline). For the subscales personal control, treatment control and coherence, higher scores represent more positive illness perceptions (e.g. more personal control or more treatment control).

Health-related quality of life was assessed by means of the Dutch version of the Quality of Life Measure for Persons with IBS consisting of 34 items (IBS-QOL) [38, 39]. The psychometric properties of the IBS-QOL were demonstrated to be good. The test–retest reliability was good after a 1-week interval. Internal consistency was found to be high for overall QOL (*α* = 0.95), as well as for all of the subscales (ranging from 0.74 to 0.93) with the exception of the subscale relationships (*α* = 0.65). Criterion validity was good as the questionnaire distinguished well between mild, moderate and severe IBS patients. Convergent validity analyses pointed out that the IBS-QOL was more closely related to overall well-being than to physical functioning measures [38]. For the purpose of the present study, the score for the total scale (*α* = 0.93) as well as the scores for the following subscales was used: dysphoria (*α* = 0.90; eight items; e.g. “I feel my life is less enjoyable because of my bowel problems”.); interference with activity (*α* = 0.85; seven items; e.g. “I feel I get less done because of my bowel problems”.); body image (*α* = 0.65; four items; e.g. “I feel fat because of my bowel problems”.); health worry (*α* = 0.37; three items; e.g. “I feel vulnerable to other illnesses because of my bowel problems”.); food avoidance (*α* = 0.73; three items; e.g. “I have to watch the kind of food I eat because of my bowel problems”.); social reaction (*α* = 0.70; three items, e.g. “I worry that people think I exaggerate my bowel problems”.); sexuality (*α* = 0.85; two items; e.g. “My bowel problems reduce my sexual desire”.); and relationships (*α* = 0.62; three items, e.g. “My bowel problems are affecting my closest relationships”.). All items were scored on a 5-point Likert scale, indicating to what extent the respondents could relate to each of the statements: not at all, slightly, moderately, quite a bit and extremely. Due to the low Cronbach *α* scores, the subscales body image, health worry and relationships were excluded from further analysis. The summed total and subscale scores were transformed to a 0–100 scale ranging from 1 (poor HRQOL) to 100 (excellent HRQOL).

### Statistical analysis

Pearson’s correlation coefficients examined univariate associations between severity of bowel symptoms (independent variable), illness perceptions (mediator variables) and HRQOL (dependent variable), as well as possible multicollinearity between the independent variable and the mediator variables. Independent *t* tests compared scores on the different HRQOL subscales between men and women, patients with a low versus high educational level and patients reporting a psychological versus somatic cause for IBS symptoms. Subsequently, six multiple mediation models were constructed and tested; each of these models placed illness perceptions as potential mediators between bowel symptom severity on the one hand and HRQOL (total score as well as the five subscale scores) on the other. Multiple mediation analysis was considered to be the most appropriate analytic strategy as it allows for multiple indirect effects to be tested simultaneously [40]. As such, the relative magnitude of the specific indirect effects associated with each putative mediator (i.e. the illness perceptions) was determined. The mediation models were tested using the bootstrapping procedure suggested by Hayes [41]. This method produces an estimate of the magnitude of each indirect effect, as well as a corresponding confidence interval. An indirect effect is assumed to be significant at an alpha level of 0.05 if its 95 % confidence interval (CI) does not include zero. A bias-corrected bootstrap CI was calculated, as this is considered to be the best test in terms of statistical power; on the basis of a number of simulations, Hayes and Scharkow [42] have demonstrated that the bias-corrected bootstrap CI is the most trustworthy test when an indirect effect exists. SPSS 20.0 was used for the descriptive and univariate analyses. The indirect.sps macro for SPSS [43] was used for all mediation analyses.

## Results

The descriptives for severity of bowel symptoms, the IPQ dimensions and the HRQOL subscales are shown in Table 1.

**Table 1:** Descriptives for severity of bowel symptoms, illness perceptions and health-related quality of life

Variable	Descriptives^a^
Frequency and severity of bowel symptoms (0–300)	168.02 ± 68.67
IPQ consequences (0–10)	6.81 ± 2.18
IPQ timeline (0–10)	9.08 ± 1.30
IPQ identity (0–10)	7.73 ± 1.61
IPQ personal control (0–10)	3.72 ± 2.16
IPQ treatment control (0–10)	4.73 ± 2.52
IPQ coherence (0–10)	6.03 ± 2.33
IPQ emotional representation (0–20)	13.04 ± 4.13
Total QOL (0–100)	61.02 ± 17.22
QOL dysphoria (0–100)	57.04 ± 22.92
QOL interference with activity (0–100)	62.54 ± 23.59
QOL food avoidance (0–100)	47.90 ± 25.13
QOL social reaction (0–100)	61.86 ± 24.33
QOL sexuality (0–100)	70.33 ± 27.09

^a^Values are mean ± SD unless otherwise indicated

No significant differences were found between men and women, patients with a higher versus lower educational level and patients reporting a psychological cause versus a somatic cause for IBS symptoms on any of the QOL dimensions.

Age was not significantly related to QOL. Severity of bowel symptoms was significantly related to both total HRQOL and each of the subscales. With respect to illness perceptions, only the Brief IPQ dimensions measuring consequences, identity and emotional representation were significantly related to HRQOL. None of the Pearson’s correlations between the independent and the mediator variables exceeded the 0.80 multicollinearity threshold suggested by field [44]. The correlation coefficients are reported in Table 2.

**Table 2:** Pearson’s correlations between age, severity of bowel symptoms, illness perceptions and quality of life (*N* = 123)

		1	2	3	4	5	6	7	8	9	10	11	12	13	14	15
1	Age	–														
2	IBSS	−0.04	–													
3	IPQ-C	0.18*	0.27**	–												
4	IPQ-T	0.12	0.07	0.07	–											
5	IPQ-I	0.07	0.46***	0.60***	0.11	–										
6	IPQ-PC	0.00	−0.13	−0.01	−0.17	−0.19*	–									
7	IPQ-TC	0.02	0.04	0.07	−0.04	0.14	0.39***	–								
8	IPQ-CO	0.10	−0.01	0.09	0.02	0.01	0.30***	0.23**	–							
9	IPQ-ER	0.04	0.38***	0.50***	0.05	0.51***	−0.04	0.01	−0.20*	–						
10	QOL-T	0.02	−0.42***	−0.68***	−0.10	−0.56***	0.13	0.04	0.00	−0.63***	–					
11	QOL-D	−0.10	−0.37***	−0.69***	−0.16	−0.56***	0.16	0.02	0.15	−0.68***	0.87***	–				
12	QOL-IA	0.04	−0.26**	−0.58***	−0.07	−0.40***	0.10	0.04	−0.08	−0.40***	0.86***	0.65***	–			
13	QOL-FA	−0.00	−0.18*	−0.38***	−0.01	−0.24**	−0.04	0.09	−0.08	−0.30***	0.61***	0.43***	0.54***	–		
14	QOL-SR	0.09	−0.30***	−0.51***	0.004	−0.35***	0.09	0.12	−0.01	−0.45***	0.75***	0.59***	0.59***	0.36***	–	
15	QOL-S	−0.08	−0.19*	−0.39***	−0.09	−0.42***	0.11	0.02	−0.14	−0.28**	0.45***	0.34***	0.38***	0.15	0.27***	–

*IBSS* irritable bowel symptom severity, *IPQ* brief illness perception questionnaire, *IPQ-C* IPQ consequences, *IPQ-T* IPQ timeline, *IPQ-I* IPQ identity, *IPQ-PC* IPQ personal control, *IPQ-TC* IPQ treatment control, *IPQ-CO* IPQ coherence, *IPQ-ER* IPQ emotional representation, *QOL* quality of life measure for persons with irritable bowel syndrome, *QOL-T* total QOL, *QOL-D* QOL dysphoria, *QOL-IA* QOL interference with activity, *QOL-FA* QOL food avoidance, *QOL-SR* QOL social reaction, *QOL-S* QOL sexuality

* *p* < 0.05, ** *p* < 0.01, *** *p* < 0.001

**Fig. 1:**
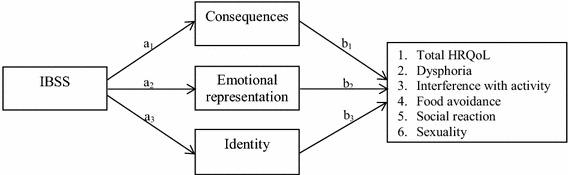
Mediation models that were tested. *Note* *a*_*1–3*_ effect of independent variable on mediators, *b*_*1–3*_ effect of mediators on dependent variables, *IBSS* irritable bowel symptom severity, *HRQol* health-related quality of life

Based on the results of the univariate analyses, only the Brief IPQ dimensions measuring consequences, identity and emotional representation were entered as potential mediators in the mediation models with severity of bowel symptoms as the independent variable and HRQOL as the dependent variable (Fig. 1).

### Mediation analyses

To answer the question whether the severity of bowel symptoms is directly and/or an indirectly related to HRQOL, mediated by the patient’s illness perceptions, we first checked the results for the direct association between symptom severity and HRQOL. IBS symptom severity was directly related to total HRQOL and each of the subscales, but after entering the mediator variables into the model, this direct relationship only remained significant (*p* < 0.05) for total HRQOL.

To explore the potential indirect association between IBS symptom severity and QOL, we first examined the relationships between the independent variable, severity of bowel symptoms, and the mediating variables consequences, identity and emotional representation of the Brief IPQ (*a* paths); the associations between the independent variable and each of the mediating variables were significant at the 0.01 level (Table 3).

**Table 3:** Summary of Mediation Analyses

Independent variable (IV)	*a* paths *a*_*n*_: IV → M	Mediators (M)	*b* paths *b*_*n*_: M → DV	Dependent variables (DV)	Indirect effect *a*_*n*_ × *b*_*n*_	95 % Bootstrap CI of indirect effect
IBS symptom severity	*a*_1_ = 0.008**	IPQ-C	*b*_1_ = −3.539***	QOL-T	*a*_1_ × *b*_1_ = −0.030**	−0.054, −0.008^a^
IBS symptom severity	*a*_2_ = 0.011***	IPQ-ER	*b*_2_ = −2.667***	QOL-T	*a*_2_ × *b*_2_ = −0.030***	−0.052, −0.015^a^
IBS symptom severity	*a*_3_ = 0.011***	IPQ-I	*b*_3_ = −0.575	QOL-T	*a*_3_ × *b*_3_ = −0.006	−0.024, 0.010
IBS symptom severity	*a*_1_ = 0.008**	IPQ-C	*b*_1_ = −4.635***	QOL-D	*a*_1_ × *b*_1_ = −0.039**	−0.074, −0.010^a^
IBS symptom severity	*a*_2_ = 0.011***	IPQ-ER	*b*_2_ = −4.558***	QOL-D	*a*_2_ × *b*_2_ = −0.052***	−0.084, −0.026^a^
IBS symptom severity	*a*_3_ = 0.011***	IPQ-I	*b*_3_ = −0.709	QOL-D	*a*_3_ × *b*_3_ = −0.008	−0.033, 0.014
IBS symptom severity	*a*_1_ = 0.008**	IPQ-C	*b*_1_ = −5.442***	QOL-IA	*a*_1_ × *b*_1_ = −0.046**	−0.088, −0.013^a^
IBS symptom severity	*a*_2_ = 0.011***	IPQ-ER	*b*_2_ = −1.327	QOL-IA	*a*_2_ × *b*_2_ = −0.015	−0.042, 0.005
IBS symptom severity	*a*_3_ = 0.011***	IPQ-I	*b*_3_ = −0.032	QOL-IA	*a*_3_ × *b*_3_ = −0.000	−0.032, 0.028
IBS symptom severity	*a*_1_ = 0.008**	IPQ-C	*b*_1_ = −3.695**	QOL-FA	*a*_1_ × *b*_1_ = −0.031*	−0.072, −0.007^a^
IBS symptom severity	*a*_2_ = 0.011***	IPQ-ER	*b*_2_ = −1.705	QOL-FA	*a*_2_ × *b*_2_ = −0.019	−0.057, 0.006
IBS symptom severity	*a*_3_ = 0.011***	IPQ-I	*b*_3_ = 0.730	QOL-FA	*a*_3_ × *b*_3_ = 0.008	−0.025, 0.045
IBS symptom severity	*a*_1_ = 0.008**	IPQ-C	*b*_1_ = −4.495***	QOL-SR	*a*_1_ × *b*_1_ = −0.038*	−0.080, −0.010^a^
IBS symptom severity	*a*_2_ = 0.011***	IPQ-ER	*b*_2_ = −2.876**	QOL-SR	*a*_2_ × *b*_2_ = −0.033*	−0.063, −0.010^a^
IBS symptom severity	*a*_3_ = 0.011***	IPQ-I	*b*_3_ = 1.177	QOL-SR	*a*_3_ × *b*_3_ = 0.013	−0.022, 0.049
IBS symptom severity	*a*_1_ = 0.008**	IPQ-C	*b*_1_ = −2.557	QOL-S	*a*_1_ × *b*_1_ = −0.022	−0.059, −0.001^a^
IBS symptom severity	*a*_2_ = 0.011***	IPQ-ER	*b*_2_ = −0.504	QOL-S	*a*_2_ × *b*_2_ = −0.006	−0.039, 0.024
IBS symptom severity	*a*_3_ = 0.011***	IPQ-I	*b*_3_ = −4.692*	QOL-S	*a*_3_ × *b*_3_ = −0.050*	−0.102, −0.013^a^

Confidence intervals (CI) presented are bias corrected and accelerated, and based on 5,000 bootstrap re-samples

*IPQ* brief illness perception questionnaire, *IPQ-C* IPQ consequences, *IPQ-T* IPQ timeline, *IPQ-I* IPQ identity, *IPQ-PC* CIPQ personal control, *IPQ-TC* IPQ treatment control, *IPQ-CO* IPQ coherence, *IPQ-ER* IPQ emotional representation, *QOL* quality of life measure for persons with irritable bowel syndrome, *QOL-T* total QOL, *QOL-D* QOL dysphoria, *QOL-IA* QOL interference with activity, *QOL-FA* QOL food avoidance, *QOL-SR* QOL social reaction, *QOL-S* QOL sexuality

^a^95 % CI does not include zero

*p < 0.05; **p < 0.01; ***p < 0.001

With respect to the association between the mediators and the dependent variable HRQOL (*b* paths), a significant relationship was found between the IPQ dimension consequences and total HRQOL, as well as the HRQOL subscales dysphoria, interference with activity, food avoidance and social reaction. The second mediator, identity, was only significantly related to the HRQOL subscale sexuality, whereas the third mediator variable, emotional representation, was significantly related to total HRQOL, and the dysphoria and social reaction subscales.

**Fig. 2:**
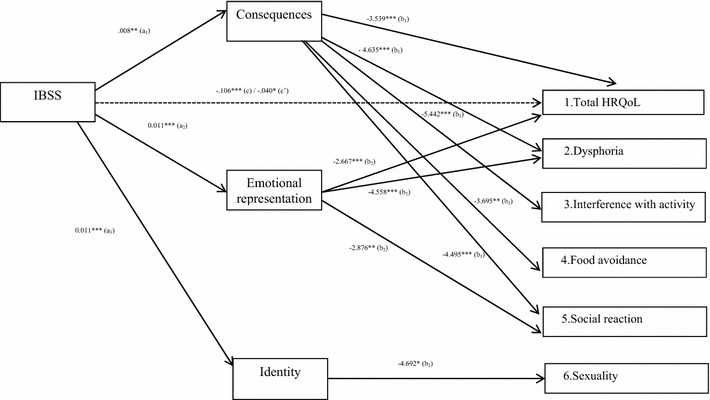
Direct and indirect effects of irritable bowel syndrome symptom severity (IBSS) on health-related quality of life (HRQOL; total and subscales) through the illness perceptions “consequences”, “emotional representation” and “identity”. Note. *a*_*1–3*_ effect of independent variable on mediators, *b*_*1–3*_ effect of mediators on dependent variables, *c* total effect of independent variable on dependent variable; c’ = direct effect of independent variable on dependent variable after the effect of the mediators was taken into account. Only statistically significant associations are depicted. * *p* < 0.05, ** *p* < 0.01, *** *p* < 0.001

Finally, the analysis of the indirect relationship between IBS symptom severity and HRQOL revealed that bowel symptom severity was indirectly related to total HRQOL as well as the subscales dysphoria and social reaction (*a* *×* *b* paths), mediated by the IPQ dimensions consequences and emotional representation. A significant indirect relationship was found between symptom severity and the subscales interference with activity and food avoidance, mediated only by the IPQ dimension consequences. The relationship between symptom severity and the subscale sexuality was only mediated by identity. The results of the mediation analyses are presented in Table 3 and Fig. 2.

As a whole, each of the models was significant, explaining 60 % of the variance in total HRQOL (Adj. R^2^ = 0.59, *p* < 0.001), 64 % of the variance in the subscale dysphoria (Adj. R^2^ = 0.63, *p* < 0.001), 36 % of the variance in the subscale interference with activity (Adj. R^2^ = 0.34, *p* < 0.001), 16 % of the variance in the subscale food avoidance (Adj. R^2^ = 0.13, *p* = 0.001), 33 % of the variance in the subscale social reaction (Adj. R^2^ = 0.31, *p* < 0.001) and 21 % of the variance in the subscale sexuality (Adj. R^2 ^= 0.18, *p* < 0.001).

## Discussion

In this study, we examined to what extent severity of bowel symptoms is directly related to HRQOL and/or indirectly, mediated by the patients’ illness perceptions.

Our main findings were: (1) severity of bowel symptoms is directly related to total HRQOL and its dimensions; (2) the relationship between bowel symptom severity and total HRQOL is partially mediated by illness perceptions, whereas its relationship with each of the separate dimensions of HRQOL is fully mediated by illness perceptions; (3) the total model, including both the independent variable disease severity and the mediators explained a very high percentage (60 %) of the variance in total HRQOL; (4) perceived consequences of IBS are a mediator of the relationship between bowel symptom severity and total HRQOL as well as all of its subscales, except one (the subscale sexuality); (5) neither perceived cause nor the control-related dimensions of the brief Illness Perception Questionnaire are significantly related to bowel symptom severity or HRQOL.

The existence of a direct relationship between bowel symptom severity and HRQOL is in accordance with previous studies [22–24]. The correlation coefficients, however, also indicate that this direct relationship is not as strong as the relationship between the psychological factors (in this case the patients’ illness perceptions) and HRQOL, which was also found by Naliboff et al. [22]. The mediation analyses that we conducted demonstrated that the relationship between bowel symptom severity and HRQOL is partially (in the case of total HRQOL) to fully (in the case of each of the dimensions of HRQOL) mediated by illness perceptions. This result somewhat fits the findings of Naliboff et al. [22], who demonstrated in a population of IBS patients that symptom severity was not only directly, but also indirectly related to HRQOL, mediated by psychological factors. The major difference between Naliboff’s study and ours is that we looked into the patients’ illness perceptions as a mediator, whereas Naliboff considered psychological distress as the potential mediating factor.

Previous studies pointed at the existence of a differential relationship between symptom severity, psychological factors and HRQOL [23, 24]. In particular, these studies demonstrated that IBS symptoms and pain were more strongly related to the physical dimension of HRQOL, whereas depression, somatic distress and anxiety were more strongly related to the psychological dimension of HRQOL. These findings are partially supported by the present study. Our results indicate that the emotional representation of illness mediates the relationship of symptom severity with the HRQOL subscale dysphoria, a disease-specific measure of depression, and, as such, a psychological dimension of HRQOL. In addition, emotional representation also mediates the association between symptom severity and the subscale social reaction, a social dimension of HRQOL that was not considered in the studies by Rey et al. [23] and Koloski et al. [24]. “Consequences”, one of the illness perceptions that underlies the cognitive representation of illness, however, mediates the relationship between symptom severity and the subscales dysphoria, interference with activity, food avoidance and social reaction, or in other words, psychological, physical as well as social aspects of HRQOL. This latter finding does not support the idea that there is a distinct pattern of associations between symptom severity, illness perceptions and HRQOL depending upon the specific dimension of HRQOL that is looked at. It rather points at the central role of the cognitive illness representation “consequences”. As “consequences” have been measured with a single item in the present study, it remains unclear which perceived consequences contribute to this finding. A number of (qualitative) studies, evaluating (the impact of) IBS “through the patients’ eyes”, [45–47] point at the fact that IBS causes multiple aspects of life to be disrupted: physical functioning, social functioning/activities/relationships/roles as well as psychological well-being. With respect to psychological well-being, patients mention feeling stigmatized by the presence of a condition for which no clear somatic cause can be found and feeling embarrassed because of the symptoms (e.g. abdominal bloating or diarrhoea). In addition, a number of misconceptions regarding the consequences of IBS are reported (e.g. the belief that IBS could lead to colitis or cancer), causing anxiety and fear in patients. Next to that, the impact IBS has on physical functioning and social activities constitutes a threat to patients’ identity and self-image and may ultimately be responsible for feelings of depression. Uncertainty about the course/chronicity of the disease and about the factors triggering symptom flare-ups and the unpredictability of symptoms may be factors underlying the disruption of patients’ lives.

The study by Rutter and Rutter [27] reported a similar relation between consequences and HRQOL but also found that attributing IBS to a psychological rather than a physical cause was associated with poorer HRQOL. Riedl et al. [29] reported that psychological illness attributions were associated with worse mental QOL, but better physical QOL, whereas somatic illness attributions were related to worse physical QOL. In our study, patients who favoured a psychological cause of IBS did not differ from patients who favoured a somatic cause on any of the dimensions of HRQOL. Differences in the way perceived cause of IBS was measured may be responsible for this. In their study, Riedl et al. [29] measured the patients’ illness attributions making use of two different questionnaires, one questionnaire to measure the dimensions “intrapsychic causes”, “social causes” and “interpersonal causes”, and one questionnaire to measure “physiological causes”; for each of these dimensions, total scores were calculated. Rutter and Rutter [27] used the “cause” item of the illness perception questionnaire [48], consisting of ten items; on the basis of a principal component analysis, two components were retained, one measuring internal psychological causes and one measuring external somatic causes. In contrast, the patients participating in our study answered an open-ended question, asking them to note down what they considered to be the most important cause of their IBS. The answers to this question were then recoded into a dichotomous variable (psychological vs. somatic causes). These clearly distinct approaches may be responsible for the difference in results.

Rutter and Rutter [27] concluded that weaker control beliefs were associated with lower HRQOL. Contrary to this finding, in our study, neither personal control nor treatment control was related to any of the dimensions of HRQOL. The way the control dimension was measured may at least be partly responsible for the differences found. In their study, Rutter and Rutter [27] used the cure/control dimension of the original IPQ [48], consisting of six items and resulting in a total score, ranging from 6 (min) to 30 (max). As such, no distinction is made between personal and treatment control in the Rutter and Rutter study, whereas in our study, two separate one-item scores are used, each of them ranging from 0 to 10.

This study has several strengths including the use of a validated disease-specific measure, the IBS-QOL [38, 39], to assess HRQOL. As symptom severity and illness perceptions are disease-specific constructs, it is important to also measure the outcome on a disease-specific level. The diagnosis of IBS was made using the Rome III criteria [2], and validated measures were used to assess bowel symptom severity [34] as well as the patients’ cognitive and emotional perceptions of the illness [36]. Furthermore, this study is also the first one to examine whether the relationship between bowel symptom severity and HRQOL is mediated by the patients’ illness perceptions. The study has, however, also some limitations. First, the study has a cross-sectional design and therefore does not allow for clear-cut conclusions regarding the direction of the relationships that were found. Based upon the basic tenet of cognitive behavioural theory, it was hypothesized that a trigger (bowel symptoms) activates cognitive processes (illness perceptions), which in turn has an impact upon emotional and behavioural factors (HRQOL). It is, however, also feasible that poor functioning (HRQOL) leads to more negative illness perceptions, which in turn influences symptom severity. Future studies should therefore adopt a longitudinal perspective to evaluate whether illness perceptions mediate the impact of IBS symptom severity on HRQOL, measured at a later time point, or whether the relationship is bidirectional. Second, in the present study, mediation models were constructed and tested for each of the six dependent variables separately. As the number of subjects available for the analyses was rather small compared to the number of models and pathways tested, this may have led to a type I error, and as such possibly to an elevation of false positives. Based on the results of our exploratory analyses into the mediators of the relationship between bowel symptom severity and HRQOL, future research should focus on constructing more parsimonious models, making use of SEM. Third, to limit participant burden, illness perceptions were measured with the Brief IPQ, consisting of six single-item scales and one two-item scale. As a consequence, two of the putative mediators in the mediation models are represented by a single-item measure (i.e. consequences and identity). With respect to this, Broadbent et al. [36] found that the correlation coefficients between the single-item scales of the Brief IPQ and the multi-item scales of the IPQ-R were highly significant (*p* < 0.001), suggesting that the single-item scales sufficiently capture the conceptual domain of each of the illness perceptions measured by the IPQ-R [49]. Nevertheless, within the psychometric literature, single-item measures have been criticized for their lack of precision, because they tend to categorize people in a relatively small number of groups [50]. Future research should therefore consider measuring illness perceptions by means of its multi-item counterpart, the IPQ-R [49]. Fourth, due to their low internal consistency, three subscales of the IBS-QOL (body image, health worry and relationships) have been excluded from the statistical analyses. As a consequence, we did not explore to what extent patients’ illness perceptions (partially) mediate the relationship between symptom severity and these three HRQOL subscales. Fifth, no data on illness duration, time since diagnosis or specific treatment followed by the patients were available to us. As these variables may have an impact on the way patients perceive their condition, this dimension should be taken into account in future research on the relationship between symptom severity, illness perceptions and HRQOL. Finally, this study was conducted in members of an IBS patient support group, which may reduce the generalizability of the results.

The main finding of the present study is that, in addition to a direct relationship, symptom severity also has an indirect relationship with HRQOL, via the cognitive and emotional representations patients have of their illness. Perceived consequences, emotional representation and to a lesser extent identity were found to be a mediator of total HRQOL and some of its subscales. There is, however, an important theoretical concern about this finding, a concern that also applies to other studies that explored the relationship between disease severity, illness perceptions and HRQOL. There is clearly a conceptual overlap between specific illness perceptions such as (a) identity and disease severity if the disease severity measure is based on patient reports and (b) consequences and emotional representation on the one hand and physical and psychological or emotional aspects of quality of life scales on the other hand. Such overlap makes it difficult to identify a clear cause–effect relationship between these concepts, since the disease severity measure may be influenced by identity perceptions, while beliefs about consequences and emotional representation may be a cause as well as a consequence of poor HRQOL and functioning. For future research, it is therefore advisable to also include measures that assess possible cognitive and behavioural coping responses that result from these perceptions but are conceptually less contaminated such as catastrophizing, rumination, generalization (cognitive responses) or limiting behaviour and symptom management (behavioural responses). This would allow clinicians wanting to improve the patient’s quality of life to focus not only on negative illness perceptions but also on dysfunctional coping patterns. In their 2012 review, Petrie and Weinman [51] discussed the central role of illness perceptions as a determinant of patient coping and patient-related outcomes and pointed out that interventions, that focus on changing patients’ illness perceptions, are an emerging field within health psychology. With respect to IBS, there is increasing evidence for the efficacy of cognitive behaviour therapy (CBT), a psychological treatment that focusses both on identifying, challenging and changing negative dysfunctional thoughts about IBS (symptoms) and on behavioural coping strategies [52]. A recent study [53] explored cognitive, emotional and behavioural mediators of treatment effects following a brief CBT intervention for IBS. It was demonstrated that cognitive factors, i.e. illness perceptions (measured with the Brief IPQ) mediated treatment effects. A limitation of this study [53] is, however, that a sum score was calculated from the Brief IPQ and entered into the mediation model. As a consequence, it remains unclear which specific illness perceptions are the most important mediators of treatment effects. In addition, the relation between specific illness perceptions (such as consequences and emotional representation) and behavioural responses was not explored. Future cognitive-based intervention studies in IBS should examine this further.
